# A comparative evaluation of three point-of-care tools by registered nurses

**DOI:** 10.5195/jmla.2022.1388

**Published:** 2022-07-01

**Authors:** Annie Nickum, Rebecca Raszewski, Susan C. Vonderheid

**Affiliations:** 1 anicku2@uic.edu, Assistant Professor & Information Services & Liaison Librarian, Library Liaison to the UIC Chicago Nursing Community, University of Illinois Chicago, Chicago, IL; 2 raszewr1@uic.edu, Associate Professor & Information Services & Liaison Librarian, Library Liaison to University of Illinois at Chicago Graduate Nursing, University of Illinois Chicago, Chicago, IL; 3 vonde@uic.edu, Clinical Assistant Professor, Director of Nursing Research, University of Illinois Chicago, Chicago, IL

**Keywords:** Point-of-care tools, evidence-based practice, evidence-based nursing, information needs

## Abstract

**Objective:**

This study compared three point-of-care tools (PoCTs) to determine which PoCT was rated highest based on key features and characteristics by registered nurses.

**Methods:**

The PoCTs reviewed were Nursing Reference Center Plus, ClinicalKey for Nursing, and UpToDate. Nurses were asked to use each PoCT to answer three clinical questions and then rate their experience based on the following areas: currency, relevancy, layout, navigation, labeling, and use of filters. They were also asked to indicate their familiarity with each PoCT, their overall opinions, and demographic information.

**Results:**

Seventy-six nurses completed the entire survey. Ratings of PoCTs did not differ by participant characteristics. Participants were most familiar with UpToDate, and average ratings were similar across all three PoCTs. Answers to open-ended questions suggested that nurses' experiences searching and locating relevant information to address clinical questions varied and that brand recognition might have impacted preference.

**Discussion:**

None of the PoCTs was significantly preferred over the others, nor received high ratings, which suggests that organizations need to survey their nurses to determine which PoCT is preferred by their staff. Findings also suggest that institutional priorities can guide the decision whether a library should license multiple PoCTs, nursing, and/or non-nursing specific PoCTs. Research is needed to understand how PoCTs could better meet the information needs of registered nurses. Librarians should learn more about what types of information nurses are seeking and explore opportunities to educate nurses on how to better utilize PoCTs for their practice.

## INTRODUCTION

Nurses are challenged to keep up with the latest in evidence-based practice, and many do not feel they are meeting standardized competencies [1]. Many barriers exist on an individual and organizational level that can limit nurses' ability to stay abreast of changes in practice: not enough time due to staffing and competing care demands, often little organizational support, and a lack of adequate training.

Point-of-care tools (PoCTs) are used by health care professionals to inform patient care at the bedside. These tools, which are often integrated into electronic health records, synthesize evidence-based information from the health sciences literature, practice guidelines, and other related resources on diseases, conditions, and procedures. In addition to their clinical utility, they also can be used to support practicing clinicians' professional development [2, 3]. Given the numerous PoCTs on the market, health care organizations and health care professionals need to decide which PoCTs offer high quality evidence in a user-friendly interface that will enable nurses to answer clinical questions at the point of need [4, 5, 6].

To date, limited research has been conducted that examines specifically whether PoCTs address the unique information needs of nurses [6]. Previous studies have predominantly focused on other health care practitioners or examined the credibility of PoCTs. The work of Banzi and colleagues [4] and the subsequent studies done by Campbell and Prorok [5, 7] found variation in content presentation across PoCTs. These studies also highlighted inconsistencies in transparency of content generation and editorial processes. A common finding was that not one PoCT met all the evaluation criteria; it was best practice to use more than one PoCT [4, 5, 7]. Further research evaluated PoCTs from the perspective of the end users with a focus on health sciences students and clinicians without discriminating between disciplines. While there were no significant differences in perception of content quality across PoCTs, the more satisfactory the user experience, the more likely the participant was to use the PoCT in the future [8, 9].

Little is known about PoCT features and characteristics from the perspective of registered nurses. Clarke [10] evaluated the information needs of both physicians and nurses. She noted that while several previous studies examined the needs of providers in general, nurses have unique information needs. Broadly speaking, physicians look at diagnoses, nurses focus more on protocols and procedures, and both look at treatment information [10]. Of the two studies focused on nurses, librarians found that nursing-centric PoCTs included more relevant nursing content than PoCTs for medical care [11], and that nurses found the content of a nursing-centric PoCT useful in clinical practice [6]. Therefore, because direct care registered nurses have their own information needs, their input is crucial when it comes to the decision-making process of choosing a PoCT.

In 2019, the nursing shared governance Coordinating Council for University of Illinois Hospital and Health Sciences System (UIH) asked that the Advanced Practice and Research Council examine nursing PoCTs and make a recommendation as to which PoCT nurses should utilize for best practice. Since 2009, University of Illinois Chicago has licensed Nursing Reference Center Plus (formerly Nursing Reference Center) from EBSCO through a package deal. Over the past five years, Nursing Reference Center Plus (NRC+) had been underutilized, making it difficult for the library to justify renewal. In addition to NRC+, the library licensed UpToDate and other PoCTs designed for clinicians. Although the council sought to evaluate other nursing PoCTs in early 2020, the COVID-19 pandemic delayed survey development and pursuing IRB approval.

In the meantime, University of Illinois Chicago nursing librarians conducted their own analysis of five PoCTs based on content, coverage of nursing topics (e.g., terms using NIC and NOC), transparency (presentation of evidence), customization (e.g., personal accounts, mobile application access), and user perception (e.g., information display, ease of use). The PoCTs examined were ClinicalKey for Nursing (CK Nursing), DynaMed, Lippincott Nursing Advisor and Procedures, NRC+, and UpToDate. They independently extracted criteria using a rubric they developed and reported descriptive statistics of the results. Their conclusions mirrored Banzi's and Prorok's: There is no one ideal PoCT; they all have strengths and weaknesses, and brand recognition is not an adequate indicator of quality [4, 7]. In looking at PoCTs from a more nursing-centric perspective, investigators determined that some PoCTs, such as UpToDate and DynaMed, were more suited to advanced practice nurses and other providers, whereas NRC+, CK Nursing, and

Lippincott focused more on nursing core measures, cultural competencies, and interventions that were more relevant to direct care nurses.

The objective of the current study was to determine which nursing PoCT would best meet registered nurses' information needs at our organization by comparing three PoCTs (NRC+, CK Nursing, UpToDate) based on clarity of content, relevance of results, currency of displayed information, ease of navigation, and their perceived familiarity. Based on our earlier study [11], we expected a nursing focused PoCT would better meet registered nurses' information needs compared to UpToDate that targets providers.

## METHODS

The authors used a quantitative descriptive design to develop an online survey that included questions that would compare NRC+, CK Nursing, and UpToDate. The survey design was based on a study by Campbell [5] with input from nursing leadership and the Advanced Practice and Research Council. Clinical questions were reviewed by a panel of sixteen clinical experts followed by minor revisions for clarity.

NRC+ and UpToDate were chosen because the library currently licensed them; a trial was obtained for CK Nursing due to interest among nursing leadership. Though there was a consensus among our study team and the organization's nursing leadership that UpToDate was geared more toward physicians and advanced practice nurses, UpToDate was included in the study because a non-nursing-focused PoCT would be the only option for nurses if the NRC+ license was cancelled or other nursing-specific PoCTs were not available.

### Setting and Sample

This study was conducted at a 462-bed university hospital in a large urban midwestern city. We used a convenience sample of 1,150 staff nurses and clinical nurse educators. This study was approved as exempt by the University of Illinois Chicago's human subjects Institutional Review Board (protocol 2020-1455).

### Recruitment and Data Collection

Participants were recruited using three strategies: emails distributed to mailing lists for registered nurses, study flyers placed in breakrooms at the hospital, and announcements about the study during monthly nursing council meetings. Participants were self-selected by following a link to the survey from an email requesting participation in the survey. No identifying information was collected from the survey itself. Participants also had the option of opening a second survey after completion of the first to enter in their names for an Amazon gift card. The participants' contact information was deleted once e-gift cards were received by participants. Data were collected from November 18, 2020, to December 31, 2020. Email reminders about the survey were sent two weeks after it opened and then two weeks and one week before it closed.

Informed consent was obtained prior to the start of the survey by including a statement indicating that proceeding with the survey implied consent. Participants were asked to choose one of three categories for clinical questions designed to simulate the experience of being a direct care nurse needing a finite answer to a patient care question: guidelines and understanding of disease, assessment and diagnosis, or nursing interventions and medication information. Once the category was chosen, nurses were instructed to search for answers to the three predetermined clinical questions using each PoCT, spending approximately three minutes per question. This short time frame was selected to simulate the clinical setting and was consistent with methods used by Campbell [5].

Survey questions to evaluate the end-user experience addressed six evaluation criteria (Table 1): clarity of content (layout), relevance of results, currency of displayed information, ease of navigation, labeling, and use of filters (see Appendix A for full survey).

**Table 1:** Evaluation Criteria for Likert Questions

Criteria	Definition
Clarity of content (layout)	Information (content) displayed on the screen was clear and concise
Relevance of results	Relevance of the results displayed was highly applicable to the clinical question
Currency of displayed information	Information displayed appeared to be the most recent available
Ease of navigation	Site was intuitive and easy to navigate
Labeling	Content was clearly labeled (e.g., headers, links)
Use of filters	The use of filters to refine the search was user friendly (e.g., age of patient)

Nurses rated their experience on a Likert scale of strongly disagree (1) to strongly agree (7). Level of familiarity with the PoCT was assessed on a scale with two end points: 0 indicating “not at all familiar” and 5 indicating “very familiar.” Other questions asked nurses whether the PoCT was previously used (yes/no), and if they used (or would use) the PoCT's mobile app (yes/no). Open-ended questions asked about their likes, dislikes, and overall opinions of the PoCTs. After participants completed their searches, they were instructed to return to the survey to answer the evaluation questions for each PoCT and complete the demographic questions detailing their area of practice, employment status, years of nursing experience, and highest level of education. The survey took approximately thirty minutes to complete.

### Data Analysis

Qualtrics, Excel, and SPSS were used for data analysis. Descriptive statistics were used to examine means, standard deviations, frequencies, and percentages. Analysis of variance (ANOVA) was used to compare overall ratings tool features and characteristics (layout, relevance, currency, navigation, labelling, filters, etc.) across the three PoCTs. A p<.05 was considered statistically significant.

Responses to open-ended questions were examined independently by the authors using inductive content analysis to identify keywords and phrases that represent themes across participants [12]. An inductive approach allowed us to remain open to additional evaluation criteria and opinions. Authors met to discuss any differences and reach consensus. Excel software was used to organize the data. More than one theme could be applied to each response. The themes, Positive and Negative, were applied to comments that were favorable or critical of each PoCT, including comments that did not identify specific characteristics of the PoCTs or their user-experiences.

## RESULTS

### Demographics

A total of 177 nurses started the survey, with 76 nurses completing the entire survey for final response rate of 6.6% of the nurses within the hospital system.

Most participants' highest level of education was a bachelor's (51%) or master's (36%) degree. Approximately 6% had an associate's or diploma from a two-year program and 7% a doctoral degree. Most participants worked in inpatient units (71%) and were full time (89%). Nursing experience ranged from zero to five years (35%), six to fifteen years (30%), or over twenty years (30%). There were no differences in nurses' ratings of the PoCTs by education level, inpatient versus outpatient, employment status, or years of experience.

### Participant Ratings

Participants were asked if they had used each PoCT previously and if so, to indicate their level of familiarity. Regarding previous use, 42% indicated they had used NRC+, 53% UpToDate, and 20% CK Nursing. Of those who had used the tool previously, Figure 1 shows their indicated level of familiarity, with 0 to 2 as not very familiar, 3 to 4 as somewhat familiar, and 5 as very familiar.

**Figure 2:**
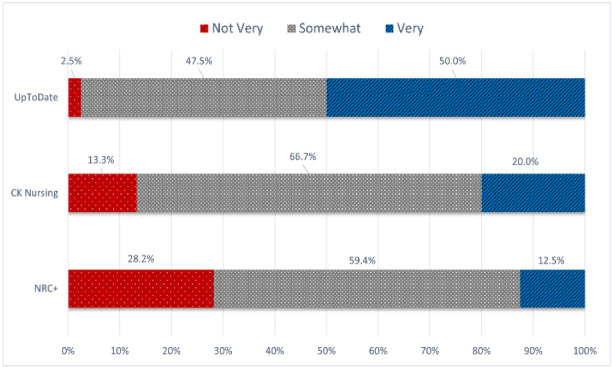
Nurses' stated familiarity with PoCTs

Responses to Likert scale ratings of the features and characteristics (layout, relevance, currency, navigation, labelling, filters, etc.) are shown in Table 2. An ANOVA comparing the overall ratings of features and characteristics found no statistical difference across PoCTs (Tool 1: x̅=6.22; Tool 2: x̅=6.19; Tool 3: x̅=5.85 (F=1,81 (2, 72) >.05)). Neither previous PoCT usage, experience, education level, nor familiarity were associated with overall ratings.

**Table 2:** Perceptions of PoCT features and characteristics. Mean and standard deviation for each PoCT are reported.

Feature of Interest	NRC+	CK Nursing	UpToDate
Layout	5.12 ± 1.5	5.3 ± 1.2	5.6 ± 1.2
Relevance	5.0 ± 1.7	5.2 ± 1.4	5.6 ± 1.4
Currency	5.4 ± 1.4	5.4 ± 1.4	5.7 ± 1.2
Navigation	5.1 ± 1.5	5.3 ± 1.2	5.5 ± 1.4
Labeling	5.4 ± 1.3	5.4 ± 1.2	5.6 ± 1.3
Filters	4.6 ± 2.0	4.5 ± 2.0	5.3 ± 1.5

### Participants' Comments about PoCTs

Fifty-five (72%) participants answered one or more of the open-ended questions. The responses were very brief— typically a couple of words or a sentence. Responses revealed the following themes representing characteristics or their user-experience of the PoCTs: Accessibility, Audience, Content, CE (Continuing Education), Current, Drugs, Familiarity, Filtering, Findability/Search, Guidelines, Info Amount, Layout, Mobile Application, Navigation, Organization, Negative, Positive, Procedures, Relevance, Simplicity, Trusted/Reliable, User Friendly, Visuals, and Other.

### Nursing Reference Center Plus

Participants provided more comments on NRC+ than the other PoCTs. When asked what nurses liked about NRC+, of the forty-eight participants who responded to this question, the most common themes were Simplicity, User Friendly, Content, Info Amount, and Navigation. Participants' favorable comments on NRC+ included, “There is a wide range of information about topics being searched” and it is a “one stop resource.” NRC+'s interface and its navigation were “easy to use.” One participant commented that it was “easy to access topics through search bar” while another mentioned “that you can download app and have all this literature at your fingertips.”

When participants were asked what they did not like about NRC+, of the thirty-eight who responded, several participants' negative comments focused on the following themes: Findability, Relevance, Info Amount, and Layout. One participant wrote they were “not able to find everything I looked for.” Another commented that the “navigation is antiquated, difficult to search, glitches when trying to filter search results,” while another wrote, “Sometimes hard to find needed info, most up-to-date things do not always reflect hospital policy.” There were also several comments within the open-ended questions that focused on struggling with finding information or sifting through a lot of information that was irrelevant to their search scenario.

### ClinicalKey for Nursing

When participants were asked what they liked about CK Nursing, of the thirty-eight who responded, the most common themes were Simplicity, User Friendly, Navigation, Content, and Findability. Unlike NRC+, however, participants commented positively on the ability to filter content within CK Nursing. Comments from various participants stated this PoCT was “easier to navigate, [had] better search results/filter use actually works” and the content “was clear and right to the point, very nursing related and specific.” A couple participants also compared CK Nursing to NRC+, such as CK Nursing was “easier to use than the first” or “This site was better than the previous. I found relevant clinical skills assessment checklists and videos/images related to each question posed.”

When participants were asked about what they did not like about CK Nursing, of the thirty-two who responded, the most common themes were Findability, Content, Relevance, and Other. Participants also had difficulty finding answers to the clinical questions within CK Nursing. One participant wrote, “The results show up like a Google search; no way to differentiate,” while another wrote, “Too many irrelevant items came up at the beginning of the search.” Another participant commented that “search did not have AND/OR option. Should add explanation/descriptor when hovering over each browse or tool option.”

### UpToDate

When participants were asked what they liked about UpToDate, of the thirty-eight who responded, the most common themes were Simplicity, User Friendly, Navigation, Organization, Content, and Findability. One participant wrote that they “love Up to Date because you are able to find the information that you are looking for relatively quick. Up to Date provides a sufficient amount of information.” Another participant commented that UpToDate “is a go-to resource” while another wrote UpToDate is “excellent for diagnosis and management of disease; knowledge of diagnosis.”

When participants were asked what they did not like about UpToDate, of the thirty-seven who responded, the most common themes were Relevance, Findability, Audience, Layout, and Organization. Participants also mentioned they had difficulty finding and searching for information within UpToDate. One participant wrote UpToDate was “text heavy, would like more differentiation in search” while another commented, “I could not find the answers in 3 minutes. I could not find a way to the site to help me find skills versus date.” Another wrote, “You have to type exactly what you want to know otherwise it doesn't seem to know what you are talking about.” Out of all three PoCTs, UpToDate received the most comments regarding its suitability for nursing, and comments were not favorable. For example, one participant wrote UpToDate is more “Medical focused, not Nursing focused,” while another wrote, “This site seemed more appropriate for diagnosing clinicians, not for nurses. The answers generated from my questions were more related to diagnosing than to nursing assessment.”

### Previously Used PoCTs and Resources

The PoCTs chosen for the survey represent only a sample of resources nurses may use to conduct evidence-based practice; therefore, participants also were asked if there were any other PoCTs or other resources they had used previously (Table 1). More than two-thirds of participants used CINAHL and PubMed, and nearly half used Lippincott Nursing products.

**Table 3:** Other PoCTs and resources respondents used previously

PoCTs and Resources	Percentage of Respondents
CINAHL	75%
PubMed	66%
Lippincott Nursing Products	41%
Cohrane Library	29%
Medline via Ovid	26%
DynaMed	13%
APA PsycINFO	9%
Other, please specify*	3%

* Other resources reported: DePaul online library search; ScienceDirect.

## DISCUSSION

The relatively even representation of opinions across the PoCTs was surprising. The authors expected that a nursing specific PoCT would be favored, as nursing PoCTs are designed specifically for practicing nurses and contain content such as care plans, procedural checklists, videos, and continuing education content. Moreover, it was expected that those familiar with PoCTs would rate them higher than those who were not. Analyses were done to determine if nursing education or experience factored into preference between PoCTs or answers to the Likert questions, but no conclusive patterns were found.

Several comments revealed a lack of understanding from some nurses about the purpose of PoCTs and how to best search and navigate them:

“Could be improved to include relevant info for bedside nurses at UIH aka protocols applicable to me.”

“Easier to filter. Fine if you're researching something, like for school or a project. But not really helpful when you're working on the floor.”

“Best research app available, free for me, easy to navigate …”

“That you can search for your topic in the form of a question”

While previous research has investigated the effectiveness of nurses' information seeking [13–16], these survey responses may indicate the need to further explore nurses' information needs and information-seeking behaviors more broadly. No PoCT received high ratings, and negative comments about searching were reported across the PoCTs. The participants in this survey seemed to expect the PoCTs' search interface to function more like Google, which could be one of their primary resources for seeking information. To improve nurses' effectiveness with PoCTs, librarians, nursing faculty, and nursing leaders may need to develop additional educational interventions that explain the situations for when nurses would use a database like CINAHL, a PoCT, or Google.

A comment that the authors found concerning was, “the nurses and doctors should all be using the same information/resources.” Health care professionals have diverse information needs depending on their level of practice and how they are interacting with other professionals. A salient point identified frequently in the literature is that because not one PoCT fulfills all the information needs of all users, more than one should be licensed [4, 7, 11]. Additional training for nurses may be necessary to highlight how nursing specific resources may be more appropriate for registered nurses' clinical practice.

Another avenue for learning more about nurses' searching behaviors is to involve vendors producing PoCTs. Vendors could make more prominent links or other mechanisms for feedback from end-users to make suggestions for improvements to PoCTs. Due to the mixed reviews regarding search results for all three PoCTs, vendors may need to adjust their search algorithms. They may need to conduct additional usability studies with nurses to discover what they are looking for and how they search for content. Participants also commented that they wanted to see images or videos within PoCTs. Vendors should consider integrating multimedia content or ensure this content can be more easily discoverable. They may also need to include a user guide more prominently within the PoCTs that would assist nurses in searching the PoCTs.

Vendors may need to examine the accessibility of their content and ensure it meets standardized accessibility license language as outlined by the Big Ten Academic Alliance Libraries [17]. Although there were several participants who commented that the screen or content was “easy to read,” there were a couple of comments regarding wanting to adjust the font size. Vendors should ensure that accessibility is embedded within the PoCTs' design. Librarians should advocate for greater accessibility and take this into consideration when selecting a PoCT for their organization.

The PoCTs rated similarly across all categories, and there were no significant differences by participant characteristics. The respondents indicated they had the most experience using UpToDate. By comparison, the lack of familiarity with NRC+ also came as a surprise, as the University of Illinois Chicago had made substantial investment in promoting this PoCT to users. For example, in late 2019, an EBSCO representative visited the hospital and demonstrated NRC+ to most inpatient units. Additionally, the authors made additional rounds on morning and evening shifts to ensure training was as comprehensive as possible. On the other hand, nursing leadership never formally endorsed NRC+ as a standard resource for best practices, which may have contributed to nurses' reluctance to use it. NRC+ was originally licensed as part of a package by the library. While resource allocations and library licensing decisions are ultimately the purview of the library, this highlights the importance of developing consensus across stakeholders when licensing a new PoCT.

UpToDate offers very little in the way of options to navigate the application such as a sidebar or dashboard; although librarians felt more navigation options would be useful, nurses did not mind the limitations. This demonstrates a growing trend in database design, the preference for a simple interface. Users often equate simplicity of use with straightforward results and do not realize that more sophisticated searches can optimize their results [18]. Likewise, while NRC+ may be widely recognized among health sciences librarians, it may not be among nurses. For example, in our study, only UpToDate and CK Nursing received mentions of being “trusted” or “reliable.” Research in the field of marketing has shown that consumers often prefer brands they are familiar with over lesser-known ones [19]. Nurses may be equating trust or the reliability of a particular resource with brand recognition. However, other reasons might be that a PoCT is considered an authoritative source because it was used during their education or is commonly used by physicians and other clinicians. The nurses at UIH may have not recognized NRC+ as a comparable resource to CK Nursing or UpToDate, which also could have contributed to its lack of usage over time. Future research is needed to more fully understand how nurses perceive PoCTs, other library resources, and to consider how librarians' perceptions of what makes a useful PoCT may differ from what nurses value as end users.

### Limitations

The sample size was relatively small. Timing of the survey may have affected participation and survey results. The survey was distributed during November and December of 2020 during the COVID-19 pandemic, and less than two months after UIH implemented a new electronic health record system. This may have led to fewer nurses having the time and capacity to complete the survey, and limits the generalizability of our results. Out of the 177 nurses who started the survey, approximately half of the participants completed it. This also limits the generalizability of the results across nurses within our organization and externally. Participants may have started the survey at work and were interrupted or decided that the effort to complete the survey outweighed the value of compensation. Self-selection might also have affected ratings. As the compensation was minimal, nurses with an interest in PoCTs may have been overrepresented and our findings overestimate the favorable ratings. The survey was developed based on the format from a previous study and the clinical questions were created by clinical experts, but the survey was not validated prior to distribution.

Since each participant completed the analysis of PoCTs in the same order, growing familiarity with PoCT usage and survey questions could have factored into the ratings of the second and third PoCTs trending higher than the first. As evidenced by Calamia et al. [20], this “practice effect” may have confounded the results by measuring familiarity with the survey rather than favorable experience with the PoCT [20]. Another potential limitation is that rather than completing a search using standardized terms, respondents generated their own search terms in response to the clinical question prompts. Without the use of standardized search terms, this study cannot determine whether satisfactory or unsatisfactory search results generated were due to the performance of the PoCT or the participants. However, allowing health care professionals to choose their own search terms is consistent with how they will use PoCTs within their clinical settings. Future research should consider randomizing the order that tools appear in a survey and tracking search terms.

Surveying users only provides one indicator of any given tool's utility and usability. The importance of ease of use cannot be overstated: if an end user must look around a resource for information, they will not use it [21]. To fully understand why one PoCT may be preferred over another, future studies of nurses' use of PoCTs should consider including aspects of user experience research design [22]. The inclusion of first clicks, eye tracking, screen recording, and other usability evaluation tools would provide a wealth of useful data [23].

## CONCLUSION

Although we previously conducted research on PoCTs, including nurses' voices was essential. Consistent with previous studies, more nurses need to be trained how to use them effectively for best practice [6,13–14]. This provides opportunities for librarians to educate nurses on best practices for searching. There also needs to be an understanding between librarians who liaise with hospitals and hospital leadership. If hospitals want PoCTs, leadership needs to communicate their importance and offer nurses opportunities for regular training at orientations, nursing unit meetings, and periodically thereafter.

Shortly after the survey closed, a report that summarized the data was shared with the Advanced Practice and Research Council and nursing leadership. Since the data was not overwhelmingly positive toward the nursing PoCTs, and nursing leadership wanted to ensure an interprofessional focus was being included within the PoCT, they decided to examine other options. They reviewed Dynamic Health from EBSCO, an evidence-based tool designed to support clinical decisions, and Lippincott's Nursing products that address clinical decision support and additional resources for nurses' professional development; ultimately, they decided to license Lippincott's Nursing products. The library decided to discontinue NRC+ in June of 2021.

## Data Availability

A cleaned, deidentified version of the dataset will be placed into the University of Illinois Chicago institutional repository, 10.25417/uic.19287809.

## SUPPLEMENTAL FILES

**Appendix A:** Nursing Point-of-Care Tools Evaluation
