# Supplementary material for: A comparative evaluation of three point-of-care tools by registered nurses
Source: J Med Libr Assoc. 2022 Jul 1;110(3):323–31. doi: 10.5195/jmla.2022.1388 (PMC9782512; doi:10.5195/jmla.2022.1388)
Supplement: Supplementary file 1 — Appendix A: Nursing Point-of-Care Tools Evaluation [file jmla-110-3-323-s01.pdf]

Appendix A: Nursing Point-of-Care Tools Evaluation

You are invited to participate in a survey that asks nurses to tell us what you think about two electronic resources for accessing clinical nursing evidence when making decisions about the care of our patients. The survey will take approximately 30 minutes to complete and has been approved by the BLINDED IRB Office (Protocol # 2020-1455).

Electronic resources (point-of-care tools) for nurses make searching for clinical evidence easier and include summaries of evidence that has already been appraised. Using these tools supports the use of research and other sources of evidence in clinical decision making and development of policies and guidelines. The nursing librarians and the Advancing Practice and Research Council developed a survey to solicit nurses' feedback to determine which point-of-care tool is the most relevant for nurses at BLINDED.

Here is a list of the tools:

- Nursing Reference Center Plus
- ClinicalKey for Nursing
- UpToDate

There are two steps to evaluating the tools.

1. Select a category. The categories are Guidelines and Understanding of Disease, Assessment and Diagnosis, and Intervention and Medication Information.
2. Search the tools to answer the three clinical questions. We created a list of clinical questions that nurses might ask. Find the answer using each tool.

After you use each tool, please complete the survey to evaluate your search experience. After you complete the survey, you will have the option to enter your name and email for receipt of a gift card. The first 25 participants will receive a $25 Amazon e-gift card. The first 100 participants will be entered in a drawing to receive a $50, $75 or $100 gift card. Your contact information will be entered into a separate survey to keep your survey responses anonymous. Participation in the study is voluntary. You may choose not to participate. If you start the study and decide to withdraw and not participate, you will not be penalized. Proceeding to the survey acknowledges that you have read and accepted the terms above.

Thank you and we look forward to receiving your feedback. For questions or concerns, please contact BLINDED.

Sincerely,

BLINDED

Do you agree to the above terms? By clicking Yes, you consent you are willing to answer the questions in this survey.
o Yes
o No

QA.1
There are several types of clinical questions you might ask when looking for information. We have divided our questions into three categories below.

Please choose a category and use each point-of-care tool to answer the questions. To simulate the clinical setting, spend no more than 3 minutes on each question.

- ◯ Guidelines and Understanding of Disease *What are hypertension guidelines? *What are the risk factors for urinary tract infections? *What is the correct procedure for inserting a catheter into an adult female? (1)
- ◯ Assessment and Diagnosis *How do you auscultate breath sounds to assess for pneumonia? *How to assess for orthostatic hypotension? *What is the correct procedure for inserting a catheter into an adult female? (2)
- ◯ Nursing Interventions and Medication Information *How to insert a foley catheter for an adult female? *What are the side effects of phenytoin? *What is the correct procedure for inserting a catheter into an adult female? (3)

QB.1
Three steps to follow for Nursing Reference Center Plus/ ClinicalKey for Nursing/ UpToDate.

Step 1: Go to Nursing Reference Center Plus/ ClinicalKey for Nursing/ UpToDate
Step 2: Look for answers to the clinical questions.

Step 3: After searching the information, please answer the following questions about your search experience by marking the appropriate number. For each point-of-care tool (PoCT), you will complete the same set of questions. This will allow you to compare the PoCTs.

QC-E.1 Have you used this PoCT before you began this evaluation?

- ◯ Yes
- ◯ No

QC-E.2 If used previously, indicate your familiarity with the PoCT.

- ◯ 0 Not familiar at all
- ◯ 1
- ◯ 2
- ◯ 3
- ◯ 4
- ◯ 5 Very familiar

QC-E.3 The layout of the information displayed on the screen was clear and concise.

- ◯ Strongly Disagree 1
- ◯ 2
- ◯ 3
- ◯ Neither agree nor disagree 4
- ◯ 5
- ◯ 6
- ◯ Strongly Agree 7

QC-E.4 The relevance of the results displayed was highly applicable to the clinical question.

- ◯ Strongly Disagree 1
- ◯ 2
- ◯ 3
- ◯ Neither agree nor disagree 4
- ◯ 5
- ◯ 6
- ◯ Strongly Agree 7

QC-E.5 The information displayed appeared to be the most recent available.

- ◯ Strongly Disagree 1
- ◯ 2
- ◯ 3
- ◯ Neither agree nor disagree 4
- ◯ 5
- ◯ 6
- ◯ Strongly Agree 7

QC-E.6 *Navigating refers to browsing or moving around the point-of-care tool to find the information you need.*

QC-E.7
The site was intuitive and easy to navigate.

- ◯ Strongly Disagree 1
- ◯ 2
- ◯ 3
- ◯ Neither agree nor disagree 4
- ◯ 5
- ◯ 6
- ◯ Strongly Agree 7

QC-E.8 Content was clearly labeled (e.g. headers, links).

- ◯ Strongly Disagree 1
- ◯ 2
- ◯ 3
- ◯ Neither agree nor disagree 4
- ◯ 5
- ◯ 6
- ◯ Strongly Agree 7

QC-E.9 The use of filters to refine the search was user friendly (e.g. age of patient).

- ○ Strongly Disagree 1
- ○ 2
- ○ 3
- ○ Neither agree nor disagree 4
- ○ 5
- ○ 6
- ○ Strongly Agree 7

QC-E.11 The PoCT has an app that you can download to your mobile device. Have you downloaded the app in the past or see yourself doing so in the future?

- ○ Yes
- ○ No

QC-E.12 Please comment on what you liked about Nursing Reference Center Plus/ ClinicalKey for Nursing/ UpToDate.

____

QC-E.13 Please comment on what you did NOT like about Nursing Reference Center Plus/ ClinicalKey for Nursing/ UpToDate

____

QC-E.14 Please provide any additional comments on Nursing Reference Center Plus/ ClinicalKey for Nursing/ UpToDate

____

Q6.2 Which of the following resources have you used in the past that were not used in this evaluation?

- ☐ CINAHL
- ☐ ClinicalKey for Nursing (formerly Mosby's)
- ☐ Cochrane Library
- ☐ DynaMed
- ☐ Lippincott Nursing products
- ☐ Medline via Ovid
- ☐ PsycINFO
- ☐ PubMed
- ☐ Other. Please specify. ________________

Q6.3 How many years have you been a nurse?

- ○ 0 - 5 years
- ○ 6- 10 years
- ○ 11-15 years
- ○ 16-20 years
- ○ 21-25 years
- ○ 26-30 years
- ○ 31 or more years

Q6.4 What is your highest level of education?

- ◯ Diploma or Associate's
- ◯ Bachelors
- ◯ Masters
- ◯ Doctor of Nursing Practice or PhD

Q6.5
How would you describe your primary unit? Please indicate if it's inpatient or outpatient.

- ◯ Inpatient
- ◯ Outpatient

Q6.6 How would you describe your position?

- ◯ Full-time
- ◯ Part-time, 50% or greater
- ◯ Part-time, less than 50%
